# Improved mechanical ventilation requires more than just increased ventilator availability: A word of caution

**DOI:** 10.1002/ctm2.43

**Published:** 2020-06-04

**Authors:** Lili Guan, Shiyue Li, Jehane Michael Le Grange, Yuqiong Yang, Rongchang Chen, Luqian Zhou

**Affiliations:** ^1^ State Key Laboratory of Respiratory Disease National Clinical Research Center for Respiratory Disease Guangzhou Institute of Respiratory Health The First Affiliated Hospital of Guangzhou Medical University Guangzhou China; ^2^ Department of Emergency Medicine, Union Hospital, Tongji Medical College Huazhong University of Science and Technology Wuhan China; ^3^ Department of Respiratory and Critical Care Medicine First Affiliated Hospital of Southern University of Science and Technology Second Clinical Medical College of Jinan University Shenzhen People's Hospital Shenzhen Institute of Respiratory Diseases Shenzhen China

A cluster of pneumonia cases with an unknown etiology were identified in China in December 2019,^1^ and later confirmed to be caused by the severe acute respiratory syndrome coronavirus‐2 and subsequently named coronavirus disease 2019 (COVID‐19). So far, the vast majority of patients are presented as mild cases, with approximately 20% of patients categorized with severe or critical illness.^2^ Patients with severe or critical COVID‐19 may develop hypoxemic respiratory failure and require respiratory support to improve hypoxia or relieve dyspnea.

Mechanical ventilators are among the most significant medical devices in the treatment of severe or critical COVID‐19 patients. About 960 000 patients in the United States will need mechanical ventilation, according to an estimation by the American Hospital Association,^3^ while a previous study estimated that there were only 62 188 mechanical ventilators with comprehensive functionalities and 98 738 mechanical ventilators with limited functionalities in the United States acute care hospitals.^4^ Currently, there is a growing global demand for ventilators in health care facilities. With the reduced availability of ventilators during the pandemic, manufacturers are working together to increase capacity and meet these clinical demands.^5^ However, simply increasing the number of ventilators is not enough, and there are other issues that need to be addressed.

Not only is the number of patients requiring mechanical ventilation increasing, but there is also a shortage of respiratory therapists or well‐trained pulmonary and critical care staff, skilled in the operation of mechanical ventilators.^6^ Additionally, medical staff from other departments, retired medical staff, or even medical students are involved in treating patients with severe or critical COVID‐19. Under normal circumstances, ventilator operators receive years of training in order to use the ventilator correctly and optimize ventilation parameters. Colombo et al^7^ investigated the ability of Italian intensive care unit (ICU) physicians to detect patient‐ventilator asynchrony using ventilator waveforms. It was suggested that experienced clinicians (working in the ICU for at least the last 3 years) had an increased sensitivity for detecting patient‐ventilator asynchrony when compared to less experienced clinicians (first‐year ICU residents).^7^ The experience and ability of medical staff have a significant impact on the effective monitoring of mechanical ventilation. If the improper use of ventilators, or problems arising during mechanical ventilation, cannot be detected and solved timeously, it may limit the efficacy of ventilatory support and lead to treatment failure. In addition, the incorrect operation could even increase the risk of infection among medical staff, thus further increasing the strain placed on healthcare systems. Therefore, personnel training remains crucial during the current crisis.

In our hospitals, the basic operating standards should be maintained through relevant training courses or online training resources for inexperienced or unskilled ventilator operators, before treating severe or critical COVID‐19 patients. This becomes an increasing challenge as more experienced ICU staff become infected and need to be replaced. Hospitals can potentially utilize telemedicine for improved convenience and thereby making it easier for well‐trained and experienced ICU staff to obtain the respiratory parameters of ventilated patients.^8^ In this way, trained staff can also guide inexperienced staff in optimizing the use of ventilators, thereby save more lives.

Due to the increasing number of different types of new ventilators, manufacturers need to ensure they supply a simple operating manual or video, available in different languages, so that all staff can rapidly comprehend the various functionalities of the individual machines.

Moreover, given the current situation, when machine errors occur, ventilator technicians may not be able to debug the ventilators as quickly. Remote monitoring software and tools should be available to adjust or troubleshoot the ventilators, in order to ensure their continued and correct use, maximizing the efficient utilization of this scarce resource. Additionally, further exploration and use of different intelligence modes (e.g., neurally adjusted ventilatory assist), which automatically adjust the parameters according to the needs of the patient, may reduce the burden placed on medical staff operating ventilators.

In conclusion, although collaboration between different countries can increase global ventilator availability and help improve the current situation, this is only the first step to solve the problem. There remain additional aspects around the use of mechanical ventilators that are affected, and that also need to be considered if we are to continue providing optimal therapy for patients requiring mechanical ventilation during this crisis.

## CONFLICT OF INTEREST

The authors declare no conflict of interest.
